# Therapeutic potential of human serum albumin nanoparticles encapsulated actinonin in murine model of lung adenocarcinoma

**DOI:** 10.1080/10717544.2022.2067600

**Published:** 2022-07-26

**Authors:** Priyanca Ahlawat, Kanika Phutela, Amanjit Bal, Navneet Singh, Sadhna Sharma

**Affiliations:** a Department of Biochemistry, Postgraduate Institute of Medical Education and Research, Chandigarh, India; b Department of Histopathology, Postgraduate Institute of Medical Education and Research, Chandigarh, India; c Department of Pulmonary Medicine, Postgraduate Institute of Medical Education and Research, Chandigarh, India

**Keywords:** Non-small cell lung cancer, actinonin, peptide deformylase, human serum albumin, folate, adenocarcinoma

## Abstract

Non-small cell lung cancer comprises 85% of the global lung cancer cases. Conventional chemotherapeutics possess certain limitations like systemic toxicity and drug resistance that requires the development of new therapeutic agents for successful treatment of lung cancer. Actinonin, a human peptide deformylase inhibitor, has demonstrated anti-cancerous properties in various leukemias and solid cancer types. However, it has limited therapeutic application because of its low bioavailability and systemic toxicity if administered in free form. This limitation can be overcome by using nano-delivery systems that will increase the therapeutic efficacy of actinonin. In the present study, human serum albumin actinonin nanoparticles were prepared using a desolvation technique and folic acid was conjugated to lysine residues of albumin for effective delivery to the lung. The lung adenocarcinoma model was established 24 weeks after intraperitoneal administration of urethane and chemotherapeutic efficacy of free as well as nanoencapsulated actinonin was evaluated. This study demonstrated anti-proliferative potential of folic acid conjugated human serum albumin nanoparticles encapsulating actinonin. The intraperitoneally administered nanoformulation exhibited sustain release profile of actinonin with longer half-life and mean retention time. The reduced dose frequency resulted in therapeutic efficacy comparable to free drug *in vivo* in terms of 100% survival and reduced tumor burden along with downregulation of epidermal growth factor receptor, folate receptor α and peptide deformylase expression in lung adenocarcinoma mice model. Therefore, actinonin encapsulated albumin nanoparticles-based therapy holds great potential as an alternative strategy to improve its anti-cancerous activity against lung adenocarcinoma.

## Background

Lung cancer is among the most aggressive carcinoma with a worldwide fatality rate of 21.5% and 13.7% in males and females, respectively (WHO Global Cancer Report, 2020). The non-small cell lung cancer (NSCLC) comprises 85% of the global lung cancer cases (WHO Global Cancer Report, 2020). Unfortunately, non-small cell lung cancer chemotherapeutics illicit systemic toxicity like hematopoietic suppression, sensory neuropathy, neutropenia, nausea, alopecia and loss of appetite, nephrotoxicity, etc (Fiala et al., 2017). Currently, plethora of identified chemotherapeutic compounds are under scrutiny for targeting hundreds of participating oncogenes against NSCLC. Unanimously, mitochondrial health remains a crucial point of cytoprotection for the cancer cells. This symbiotic relationship between the organelle and nucleus governs a delicate balance of anti- and pro-apoptotic fate of the proliferating tumor cells. Also, oxidative state in mitochondria results in somatic point mutations in rRNA, ETC complexes and ATPase (Matsuyama and Suzuki, 1968). Such mutations compel cells to increase the rate of glycolysis, lactate production and HIF-1α which helps in tumor progression under hypoxic conditions. Mitochondrial translation has also been explored as a potential target to obliterate non-small cell lung cancer (Jia et al., 2016; Kuntz et al., 2017). Interestingly in 2000s, human peptide deformylase was identified for its mitochondrial presence (Giglione et al., 2000). Although its inhibition is well documented in variety of bacterial infections like of mycobacteria, chlamydia, helicobacter, staphylococci among many others to be beneficial in case of antibacterial resistance as novel target (Fritsche et al., 2005; Sharma et al., 2009; Hoover et al., 2016; Rampogu et al., 2018; Gokhale & Telvekar, 2021). However, its overexpression in various carcinoma prompted investigations on its inhibition to demonstrate its anti-cancerous potential (Randhawa et al., 2013; Sheth et al., 2014). Actinonin, an actinomycotic compound with its active hydroxamate group binds to peptide deformylase and chelates divalent prosthetic group which is essential for its catalytic activity (Chen et al., 2000). Actinonin has been studied in various leukemia and solid cancer types *in vitro* as well as *in vivo* and has been shown to be an efficacious chemotherapeutic compound (Lee et al., 2003; Lee et al., 2004; Sheth et al., 2014). However, it has some other systemic targets as well. It inhibits metalloproteases whose crucial balance maintains the tissue integrity and therefore, actinonin’s free and frequent administration can invariably affect systemic homeostasis. Also, its bioavailability is greatly reduced when administered in the free form (Sangshetti et al., 2015).

Nanomedicine has been extensively tested experimentally against non-small cell lung cancer in various *ex vivo* and *in vivo* studies (Viswanadh et al., 2020; Ghosh et al., 2021). Recently nab-paclitaxel, an albumin conjugated paclitaxel is being clinically prescribed against many solid tumors like breast, lung, etc (Langer et al., 2021; Yuan et al., 2021). Noticeably, albumin is a desirable choice of polymer for nanoparticles development as it has biological abundance which makes it nontoxic to the system and does not illicit any abnormal immune responses unlike metals and synthetic polymers. Folate is widely popular as a tumor targeting moiety as folate receptors are abundantly expressed by cancer cells (O'Shannessy et al., 2012; Rosch et al., 2020). Studies indicate that folate receptor-α can be used for targeted delivery of therapeutics (Driver et al., 2016; Moore et al., 2017; Orellana et al., 2017). As primary requisites of a desirable delivery system development comprise minimum use of synthetic chemicals, employing biologically nontoxic polymers and active targeting conjugates which provide increased bioavailability of the drug and reduced toxicity. Thus, the efforts were made to assess the therapeutic potential of folate conjugated actinonin-albumin nanoformulation for improving its bioavailability, evading its off-target effects as well as increasing its tumor site targeting.

## Methods

### Procurement of animals

Inbred BALB/c mice of either sex (8–10 weeks old, 20–25 g body weight) were obtained from the Institutional Small Animal Facility of Postgraduate Institute of Medical Education and Research, Chandigarh. The study was approved by Institution Animal Ethics Committee (IAEC) vide no. 80/IAEC/502 and 99/IAEC/692. The mice were housed in ethically approved conditions with 12 h night and day cycle and were fed standard diet and water *ad libitum.*

### Preparation of human serum albumin nanoparticles

The nanoparticles were prepared using the desolvation technique (Li et al., 2011). Human serum albumin (Sigma-Aldrich, USA) solution (30 mg/ml pH 9) was prepared. 1 mg actinonin (Sigma-Aldrich, USA and Cayman, USA) was mixed with 6 ml absolute ethanol and was added dropwise with 1 ml/min flow rate to albumin solution kept under constant stirring until turbid. 3% glutaraldehyde (Sigma-Aldrich, USA) was added as stabilizing (cross-linker) agent. Next, ethanol was completely air evaporated at room temperature, and nanoparticles suspension was centrifuged at 20,000 × g for 20 min at 4 °C.

### Folate conjugation of developed human serum albumin nanoparticles

Folic acid (HiMedia Laboratories, India) was modified and conjugated to lysine residues of human serum albumin nanoparticles. For this, 1 g folic acid was mixed with 340 mg dicyclocarbodiimide (HiMedia Laboratories, India) and 520 mg N-hydroxysuccinimide (Sigma-Aldrich, USA). 500 µl triethylamine (SRL, India) and 20 ml anhydrous DMSO (Merck, USA) were added to the mixture (Guo and Lee, 1999). The dicyclourea as byproduct was filtered and washed with anhydrous diethyl ether + 30% acetonitrile (Merck, USA) and the resulting precipitate was lyophilized. A solution of 3 mg of lyophilized activated folate (i.e. conjugate of folate-NHS) in 0.1 M bicarbonate buffer (pH 10) was added dropwise to the prepared nanoparticles suspension at 1 ml/min flow rate and kept for overnight stirring in dark at room temperature. The resulting folate conjugated actinonin albumin nanoparticles were harvested by centrifugation at 20,000 × g for 20 min at 4 °C.

### Characterization of drug-loaded folate conjugated albumin nanoparticles

The samples of folic acid, N-hydroxysuccinamide, folate-NHS, free actinonin, empty folate conjugated nanoparticles and actinonin loaded folate conjugated albumin nanoparticles were subjected to FTIR analysis. The nanoparticles’ structure was studied by the X-ray diffraction patterns collected using X-ray diffractometer at the scanning rate of 5 °C/min with diffraction angle at 0–80˚. A diluted nanoformulation was measured for particle size, polydispersity index and zeta potential (Zetasizer, Malvern Corp., USA). The morphology of the developed nanoformulation was observed using scanning electron microscope (SEM) and transmission electron microscope (TEM). SEM was done at 30 kV, 300 nm resolution in high vacuum mode (Hitachi H3400N, Japan) and TEM was performed at 60–100 kV using a JEOL microscope (JEOL JEM1400+). The amount of folate conjugated on the surface of human serum albumin after drug encapsulation was estimated by absorption at 370nm.

The drug-loaded nanoparticles were washed and total drug content was quantified using RP-HPLC (UHPLC Thermofisher, USA). Actinonin was estimated using 10 mM potassium phosphate buffer (pH 2.5): ACN = 72:28(v/v) as mobile phase with a run time of 10 min at 208 nm. The C18 column was used for the drug identification and quantification. Drug loading capacity was quantified as the amount of drug (mg) entrapped per mg of the formulation developed. The percentage drug entrapment efficiency was calculated as: [Amount of drug (mg) encapsulated in the nanoparticles]/[Amount of drug (mg) initially taken] × 100.

### Cellular uptake and effects of folate conjugated actinonin nanoparticles

NCI-A549 cell line (NCCS Pune, India) was treated with 250 μM of actinonin nanoformulation for 48 h and cellular uptake was studied using TEM. Treated cells were pelleted and kept in filtered 3% glutaraldehyde in 0.2 M Sorenson’s phosphate buffer overnight in 4 °C. Later cell pellets were embedded in EPON mixture and were kept for polymerization at 60 °C overnight. The embedded cell pellets were sectioned as semithin sections and visualized under a light microscope stained with toluidine blue. The embedded cell pellets were sectioned using ultramicrotome, stained with uranyl acetate and lead citrate before visualization under a transmission electron microscope. The samples were visualized at 60–100 kV using a JEOL microscope (model no. JEOLJEM1400+)

### Pharmacokinetic studies

Inbred BALB/c male mice (25–30 g) were administered 150 mg/kg single dose of free and nanoformulated actinonin with 6 mice in each group. Blood was withdrawn from retro-orbital plexus at 10 min, 20 min, 35 min, 50 min, 1 h, 6 h, 9 h, 24 h, 2d, and 3d from the animals administered free actinonin i.p. From the nanoformulation administered animals, blood was withdrawn at 1 h, 4 h, 6 h, 9 h, 24 h, and 48 h till 14 days. Blood was withdrawn from at least 3–4 animals at each time point. Serum was separated by centrifugation at 3000 rpm for 15 min. Also, mice were sacrificed at 24 h by cervical dislocation and actinonin levels were measured in tissue homogenates which were prepared using tissue homogenizer, centrifuged at 10,000 × g for 30 min at 4 °C. The protein-free filtrates were prepared by addition of equivolume 100% acetonitrile and drug estimation was performed using RP-HPLC. The various pharmacokinetic parameters were calculated using R and WinNonlin software based on trapezoidal rule.

### Development of murine lung adenocarcinoma model

The inbred BALB/c mice of either sex (8–10 weeks old, 20–25 g b.wt.) were administered urethane (Sigma-Aldrich, USA) at 1 g/kg b.wt. in saline once a week for four consecutive weeks intraperitoneally (Li et al., 2013). Mice were sacrificed by cervical dislocation at 8th, 16th and 24th week to track the development of lung adenocarcinoma lesions by histopathological analysis. Furthermore, the tumor development was confirmed by immunohistochemistry for Cytokeratin 7, Napsin-A and TTF-1(Cell Marque, USA) proteins as markers of lung adenocarcinoma.

### Therapeutic efficacy studies

Inbred BALB/c mice bearing lung adenocarcinoma induced by administration of urethane (i.p) were primarily divided in two major groups based on 6 and 10 months latency periods (40 weeks latency period was included as a model of advanced disease). The animals (*n* = 6/group) were further divided based on the type of therapy (free or nanoformulated drug) both administered intraperitoneally. As per pharmacokinetic profile, free actinonin was administered at a frequency of five times whereas nanoformulation for two times both intraperitoneally for one week. The doses for free and nanoformulated actinonin was 150 mg/kg b.wt. At 30th day post therapy, mice were sacrificed. The lungs tumor lesions were counted after excision and tumor size was measured manually using Vernier calipers. The lesion size was calculated from the tumor volume: (Tumor volume (mm^3^) = 0.5 (longer axis x shorter axis) (Fiala et al., 2017).

### Histology and immunohistochemistry

The organs were fixed in 10% buffered formalin. Tissues were sectioned using microtome and were placed on the albumin-coated glass slides for staining with hematoxylin and eosin. They were visualized under a light microscope (Olympus, USA). Sections were incubated with TTF-1 primary antibody for 60 min at room temperature (RT) and washed with buffer (TBS/PBS). Secondary antibody was added and incubated at RT for 40 min. Hematoxylin was added as a counter stain. Finally, sections were mounted with DPX and visualized under light microscope.

### Cellular assays of tumor lesions using singe cell suspension

The tumor lesions were excised precisely, avoiding the peritumoral regions. The excised lesions were carefully minced using glass slides. The minced tumor lesions were gently syringed 2–3 times in a graded fashion viz. 21 G→22G→24G →26 G in 1× PBS. Cells were resuspended in 1× PBS and cell suspension was divided for subsequent cytometry-based assays. The single cell suspensions of tumor lesions and control lung tissue were incubated with EpCAM-FITC monoclonal antibody (Invitrogen, USA) at 4 °C and 5 µl of CD45-PerCP antibody (BD Biosciences, USA) at 37 °C. The incubated cells were washed with 1× PBS and quantified for FITC and CD45-PerCP positive populations individually using flow cytometer (FACS Caliber, BD, USA) sanctioned by Department of Science and Technology under DST FIST project (SR/FST/LSI-584/2013).

#### Apoptosis assay

The cell pellet was mixed with 100 µl of 1× binding buffer (provided with Beckman Dickson, USA kit) to prepare cell suspension. Fluorescein isothiocyanate–labeled annexin V and propidium iodide were added and cells were incubated at RT. Cell population positive for individual and dual staining were quantified. Signals for FITC-annexin V and propidium iodide were quantified in FITC and PE band filters, respectively, by flow cytometer.

#### Reactive oxygen species estimation

The cell suspensions from *in vivo* lesions of treated and untreated mice were treated with 5 μM of DCFDA dye (Sigma-Aldrich, USA). Cells were incubated and quantified for fluorescence with excitation/emission wavelength of 490/525 nm by flow cytometry.

#### Mitochondrial potential estimation

The single cell suspensions were subjected to treatment with potentiometric tetramethylrhodamine methyl ester (TMRM) dye as per manufacturer’s instructions (Invitrogen, USA). Cells were quantified directly using PE bandpass filter in a flow cytometer.

### Gene expression analysis

RNA isolation was performed in tumor lesions and control lung section using TRIzol (Ambion Inc., Austin) based Phenol-chloroform method as per the manufacturer's instructions. cDNA was synthesized using PrimeScript 1 Strand cDNA synthesis kit (TaKaRa Bio Inc., Japan). The primer sequences of different genes were *EGFR* (Forward) TTCTTTTCCTCCAACGC, (Reverse) CCGTCTGTCTCGGATTA; *FOLR1* (*Forward*) TGGTCGTGTAAATTGTCCT, (Reverse) GGACTGAACTTCTCAATGTC; *PDF (Forward) CTGCTGTAATCGTATTCTGT, (Reverse) GGTGAATGACTAACACTCTC*; Internal control used was *RNA18S* (Forward) GCAATTATTCCCCATGAACG, (Reverse) GGCCTCACTAAACCATCCAA.

### Statistical analysis

Mann–Whitney, Kruskal–Wallis and one-way ANOVA tests were used to determine the level of significance by using SPSS software and GraphPad Prism Package version 8.0 (GraphPad Software, San Diego, USA). Pharmacokinetic parameters were calculated using R package PKNCA (Pharmacokinetic non-compartmental analysis) version 0.9.4. The R analysis was confirmed by WinNonlin Software.

## Results

### Characterization of folate conjugated human serum albumin nanoparticles encapsulating actinonin

**Figure 1.**
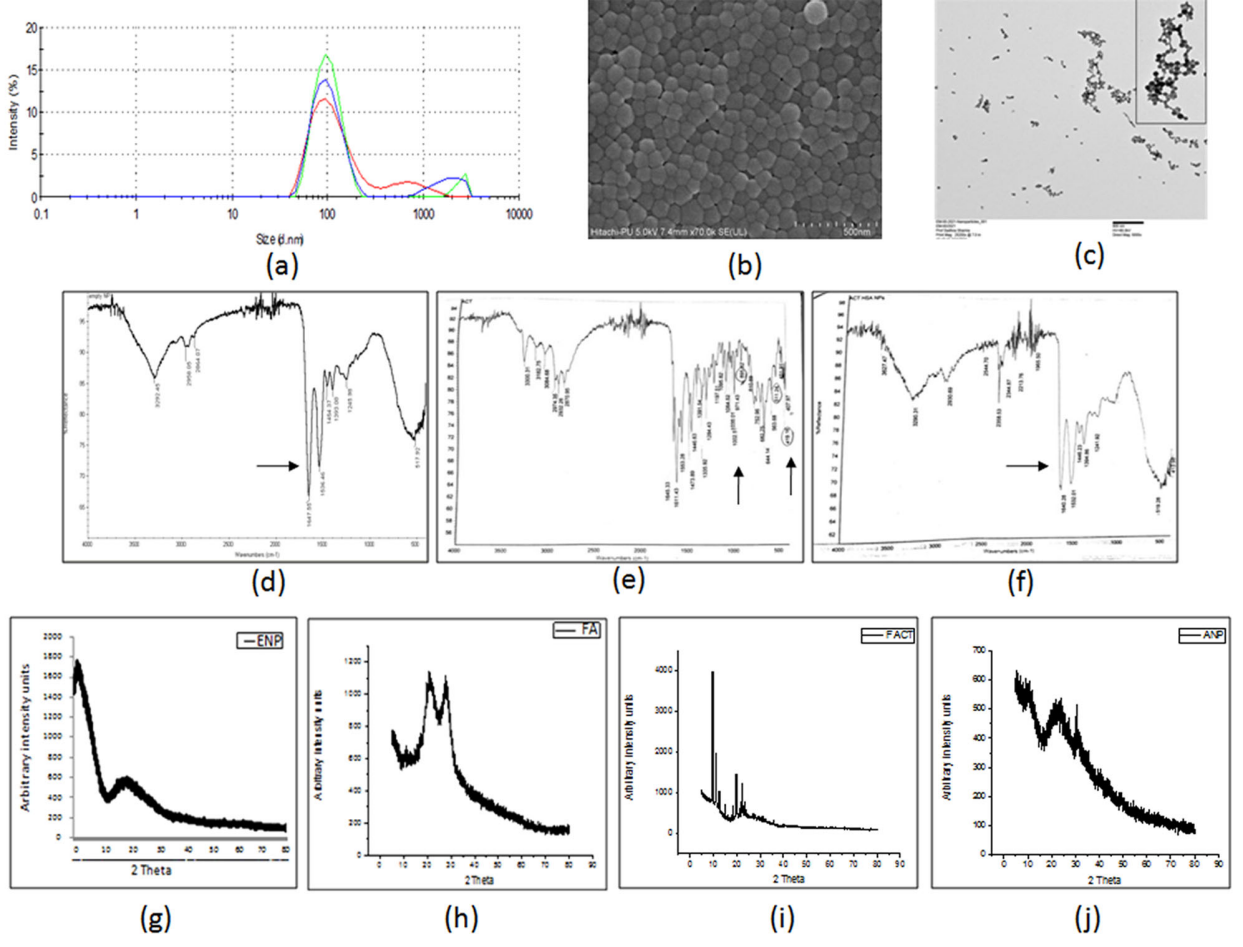
The characterization of developed actinonin nanoparticles. (a) Representative size distribution of actinonin nanoparticles. The three independent formulations are represented in three different colors. (b)Scanning electron microscopic images of actinonin nanoparticles at 70k X magnification. (c) Transmission electron microscopic image of folate conjugated actinonin nanoparticles. The FTIR data of (d) empty HSA nanoparticles, (e) free actinonin, (f) actinonin nanoparticles. The x-ray diffraction analysis of free drugs and nanoformulated drugs. (g) ENP-empty nanoparticles without folate, (h) FA-folic acid-NHS conjugate, (i) F ACT- free actinonin, (j) ANP- actinonin nanoparticles, The y-axis shows the arbitrary intensity units and x-axis – 2θ˚ (angle in degrees). The experiments were performed in triplicate.

The successfully developed nano-formulation of folate conjugated actinonin was characterized for their size using the dynamic light scattering (DLS) technique. The mean hydrodynamic size of actinonin nanoformulation was 167.4 nm (Table 1 and Figure 1). The polydispersity index and zeta potential implied that the nanoparticles were uniformly sized and evenly dispersed in the suspension form (Table 1). The amount of folate conjugated to the lysine residues of human serum albumin of pemetrexed nanoparticles was in the range of 76% (Table 1). The nanoparticles showed drug encapsulation of 60%, as estimated using RP-HPLC (Table 1). The scanning and transmission electron microscopic images clearly revealed the spherical structure of the folate conjugated nanoparticles (Figure 1b,c). Initially, folate activation was analyzed by FTIR. The NHS conjugated folic acid showed peaks at 3248.0 cm^−1^ (primary aliphatic amine N–H stretching); 2933.62 cm^−1^ (carboxylic acid C–O and O–H stretching unconjugated); 1599.0 cm^−1^ (ketones C–O unconjugated stretch); all of which confirmed the synthesis of NHS ester of folic acid. After successful folate activation, the FTIR spectra of the folate conjugated serum albumin nanoparticles showed the peaks at 2958 cm^−1^, 1647 cm^−1^, 1532 cm^−1^ and 871 cm^−1^ (Figure 1d–f). The actinonin was also encapsulated in the nanoparticles as indicated by masking of peaks at 2974.35 cm^−1^, 2870.0 cm^−1^, 1197.51 cm^−1^, 1035.01 cm^−1^ and 971.43 cm^−1^ (Figure 1f). Therefore, FTIR spectra analysis confirmed the successful folate conjugation and encapsulation of actinonin in developed human serum albumin nanoparticles. X-ray diffraction, a sensitive technique to identify the arrangement of atoms of a given nanoformulation showed wide peaks indicating amorphous structure of nanoparticles encapsulating the actinonin with the size of ultra-small scale i.e. nano meters (Figure 1i,j).

**Table 1.** Characterization of actinonin nanoparticles.

Parameters	Actinonin nanoparticles
Mean size(nm)	167.4 ± 70
Mean polydispersity index	0.28 ± 0.03
Mean zeta potential (mV)	−24.3 ± 0.15
% Encapsulation efficiency	60 ± 2.70
% Drug loading capacity	3.05 ± 2.10
Conjugated folate concentration (mg/ml)	2.30 ± 0.01
Percentage folate conjugation to the nanoformulation	76.10 ± 3.86

Values are mean ± S.D of three independent formulations.

### Pharmacokinetics studies on free and nanoformulated actinonin

Healthy inbred BALB/c mice were used for conducting pharmacokinetic analysis of free and nanoformulated actinonin.

The pharmacokinetic profile revealed prolonged retention and sustained release kinetics of nanoformulated actinonin when administered intraperitoneally. The free actinonin was detected in serum for relatively short duration as seen by lower mean retention time (MRT) and early half-life than its nanoformulation (Table 2). Also, high levels of free actinonin were present in liver and spleen than in kidney (data not shown). The therapeutic efficacy of the developed nanoformulation was tested in an experimental model of murine lung adenocarcinoma. Initially, a pilot study was conducted to establish the latency period of urethane-induced lung adenocarcinoma in mice. Inbred BALB/c mouse strain was used for the animal studies as they have intermittent susceptibility to develop urethane-induced lung adenocarcinoma.

**Table 2.** The serum pharmacokinetic parameters after single dose administration of free and nanoformulated actinonin.

Parameters	Actinonin free i.p	Actinonin nano i.p
*T*_½_ (h)	0.683 ± 0.06	87.13 ± 0.04
Mean retention time(h)	0.72 ± 0.07	133.82 ± 0.41
*K*_el_	7.5519 ± 0.01	0.008 ± 0.001
*C*_max_ (µg/ml)	10.95 ± 0.013	0.41 ± 0.01
AUMC (µgh^2^/ml)	5.413 ± 0.6	11576.15 ± 0.3
Clearance (mg/l)	463.45 ± 0.2	54.73 ± 0.13

Values are mean ± SD of 3 animals.

### Establishment of murine lung adenocarcinoma model

**Figure 2.**
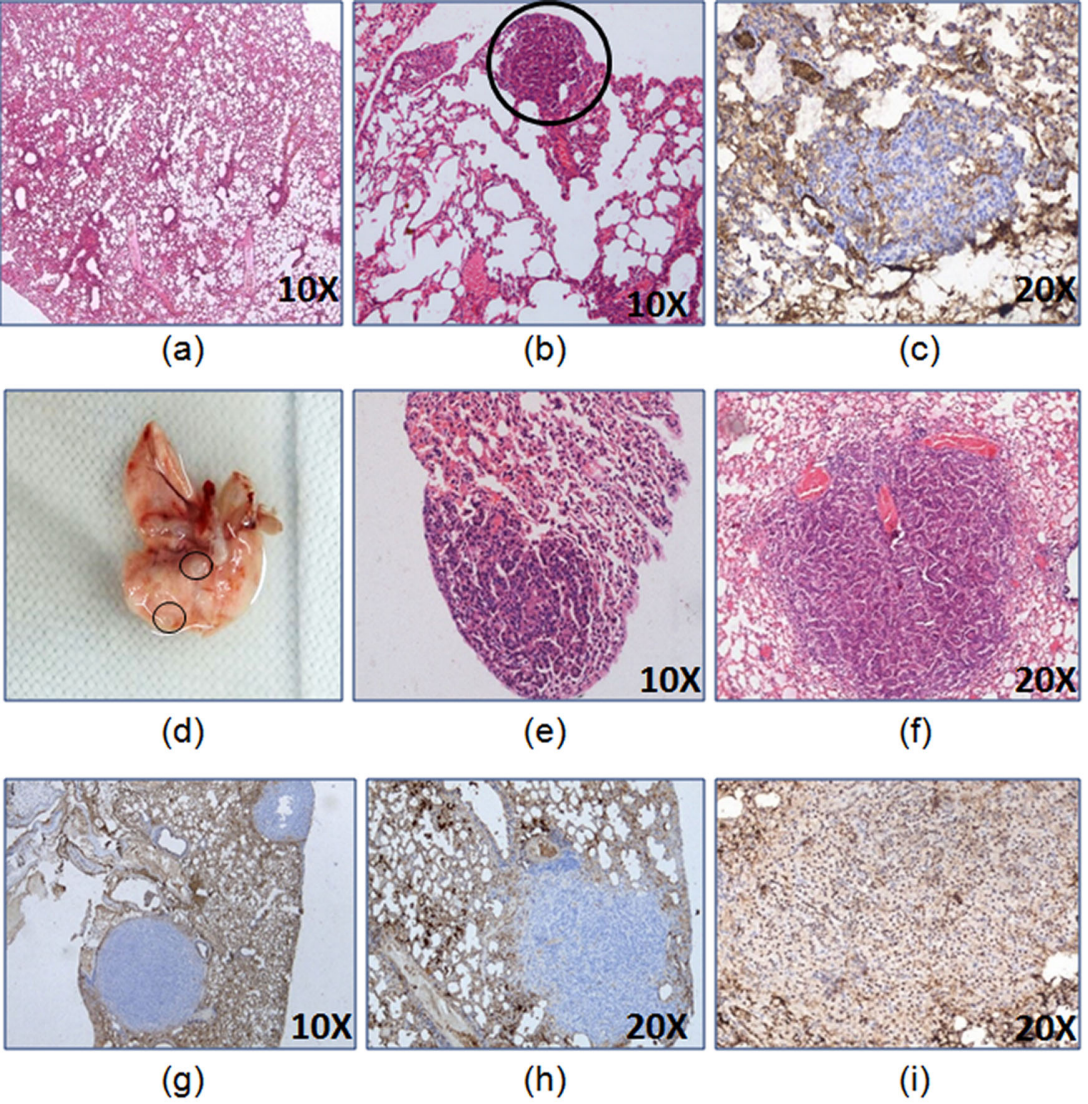
Development of lung adenocarcinoma 24 weeks model. The representative images of lungs (a) control, (b) H&E of tumor lesion at 8th week of latency, (c) CK7^-^ 8th week of latency tumor lesion, (d) gross tumor lesion at 16th week of latency,(e) H&E of tumor lesion at 16th week of latency, (f) H&E of lung tumor lesion at 24th week of latency, (g) CK7^-^ 24th week of latency tumor lesion, (h) Napsin A^-^ 24th week of latency tumor lesion, (i) TTF-1^+^24th week of latency tumor lesion.

**Figure 3.**
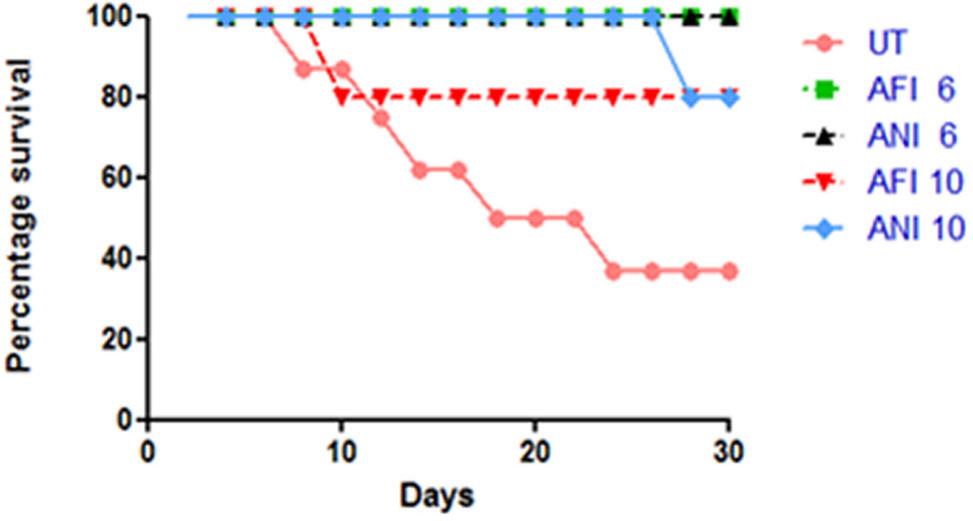
Survival analysis. The survival chart for free and nanoformulated actinonin-treated groups for both latency periods. UT – untreated, AFI – free actinonin i.p and ANI – nanoformulated actinonin i.p. 6 and 10 indicate the latency months.

As per the preliminary studies, the latency period of two months was able to form lung lesions displaying proliferating cells (Figure 2b, c) but by 6 months, mature lung adenocarcinoma was developed which was confirmed by TTF-1 nuclear positivity (Figures 2f, i and 3).

Considering the pilot study results, the therapeutic efficacy study was conducted in the mice grouped in two types of latency periods i.e. of 6 months (24 weeks) and 10 months (40 weeks). The extended period of 10 months latency was used in the study as urethane-induced carcinogenesis involves inflammation and longer duration further facilitates the enhanced tumorigenic environment. This may result in metastasis or other complications that may reflect the advanced stage of lung adenocarcinoma.

### Therapeutic efficacy of free and nano encapsulated actinonin against lung adenocarcinoma

Next, the free and nanoformulated actinonin were tested for their therapeutic efficacies and pharmacokinetic parameters were taken into consideration to decide the dose frequencies. The lung tumor-induced mice were used for therapeutic efficacy studies at 6 and 10 months latency period. The mice were subjected to a treatment regimen as mentioned in the “Methods” section.

### Survival analysis

The animals were observed for 30 days post-therapy and survival was analyzed for each group (Figure 3).

### Tumor burden

**Figure 4.**
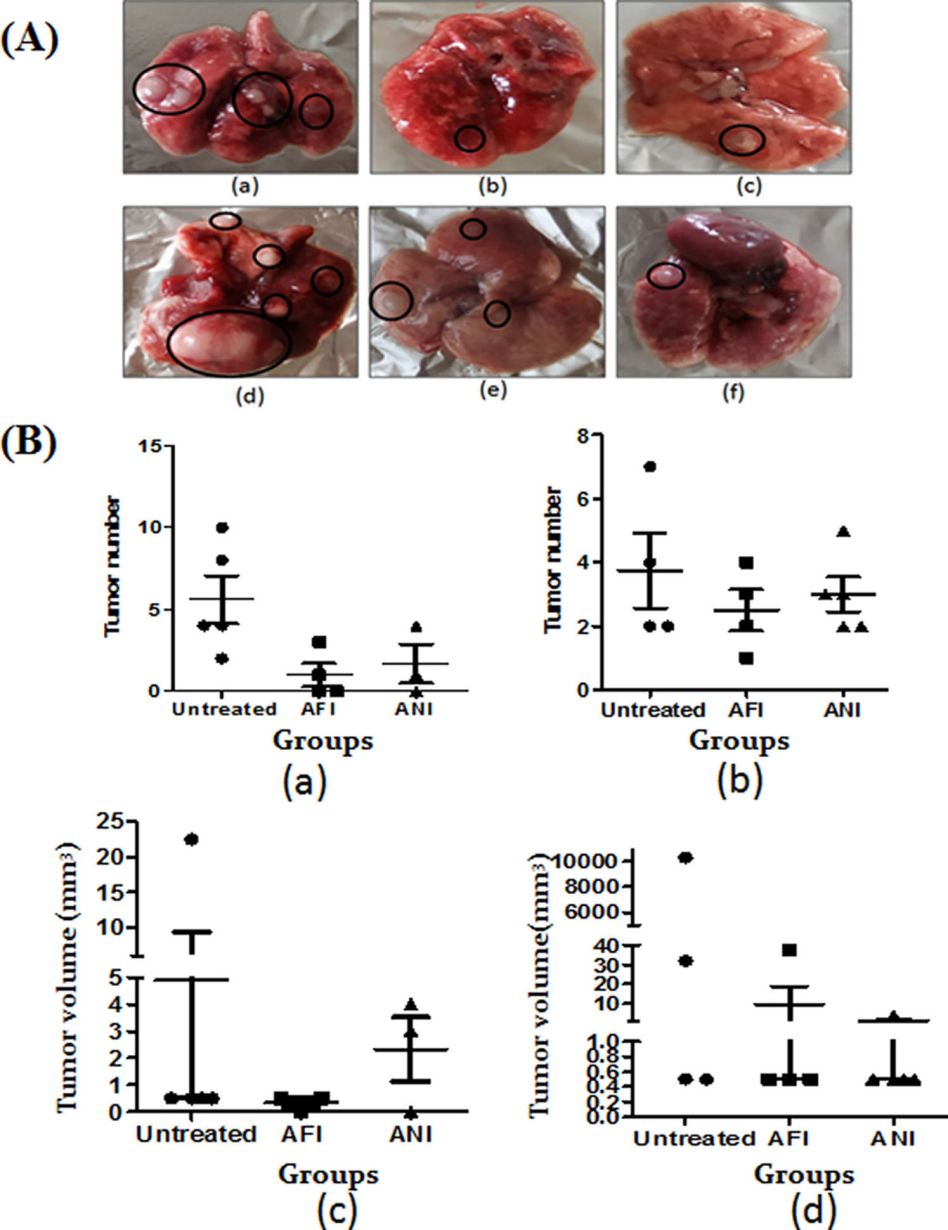
Gross lung analysis. (A) Representative gross lung tumor lesions of (a) untreated group; (b) free actinonin-treated group; (c) nanoformulated actinonin-treated group; at 6 months latency period. Representative gross lung tumors of (d) untreated group; (e) free actinonin-treated group; (f) nanoformulated actinonin-treated group at 10 months latency period. (B) The tumor number for (a) 6 months latency period groups and (b) 10 months latency period groups. The tumor volume for (c) 6 months latency period groups and (d) 10 months latency period groups. UT – untreated, AFI – free actinonin i.p and ANI – nanoformulated actinonin i.p. Circles highlight the tumor lesions. Values are mean ± S.E. of 3–5 animals.

It was evident that in the untreated 6- and 10-month latency mice groups, an average of 5–7 tumors were present (Figure 4(A)a,d). Upon treatment with free and nano actinonin administered intraperitoneally, the tumor numbers and volume showed reduction in both latency period models as compared to the untreated group (Figure 4(A)b,c,e,&f and (B)).

**Figure 5.**
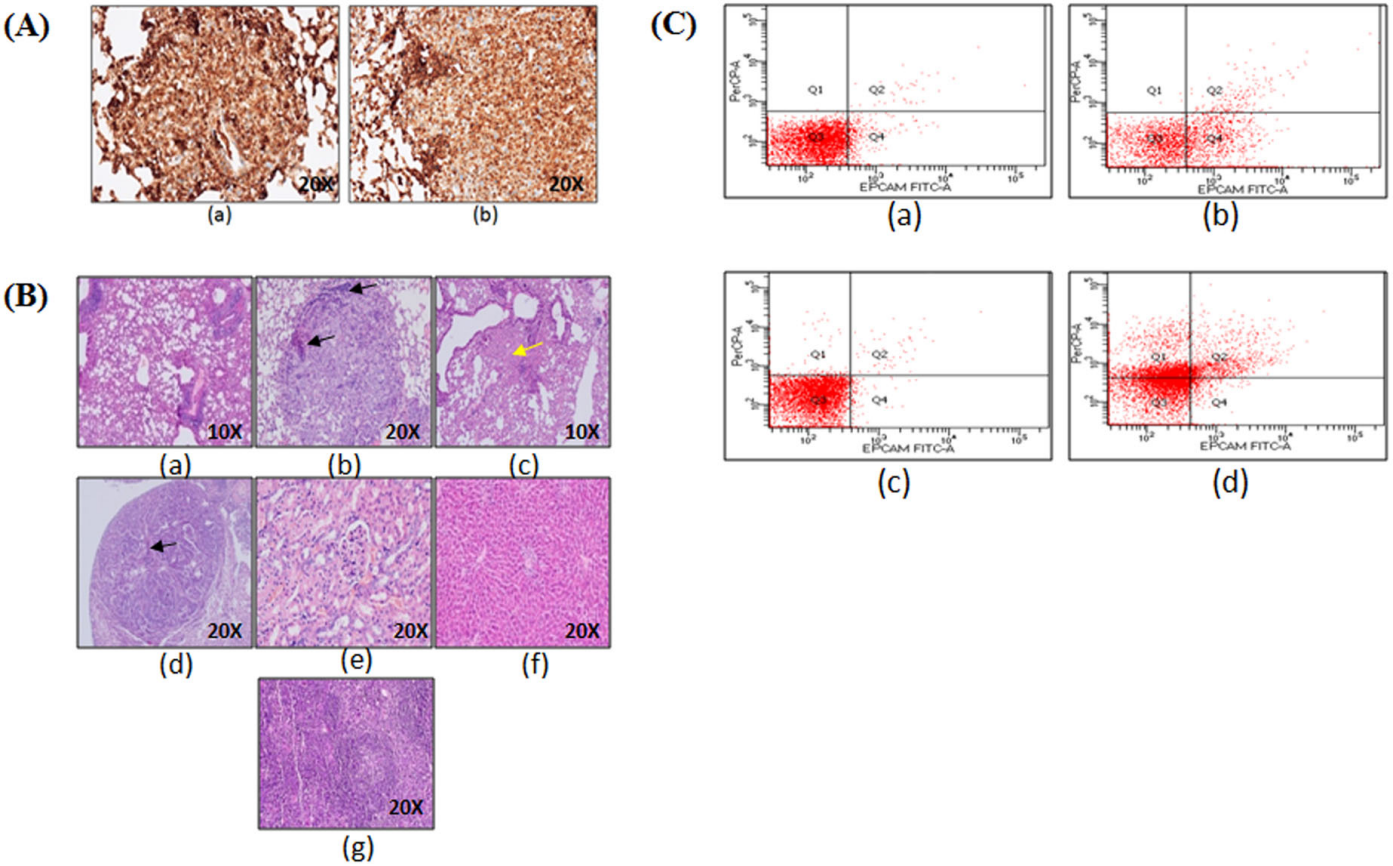
Immunohistochemistry and flow cytometric analysis of lung adenocarcinoma lesions. (A) Representative images of TTF-1^+^ lung tumor lesions at (a) 6 months latency period and (b) 10 months latency period. (B) Representative H&E lung tumor lesions of (a) control group; (b) untreated group; (c) free actinonin-treated group; (d) nanoformulated actinonin-treated group. H&E of other vital organs (e) kidney, (f) liver and (g) spleen. Black arrows indicate lymphocytic infiltration and yellow arrow indicate apoptosis.(C) The excised tumors screened for presence of EpCAM and CD45 positive cells. (a) Control EpCAM^+^, (b) Tumor EpCAM^+^, (c) Control CD45^+^, (d) Tumor CD45^+^.

Additionally, immunohistochemistry of lung tumor lesions formed after 6 and 10 months showed TTF-1 nuclear positivity thus confirming them to be lung adenocarcinoma (Figure 5(A)).

The histological observations revealed control lung with normal lung parenchymal cells. In tumor bearing mice lungs, the tumor nodules had presence of tumor cells in papillary and glandular pattern which represents classical morphological features of adenocarcinoma (Figure 5(B)b). The treatment groups with tumor nodules had few pink regions representing the apoptotic areas (Figure 5(B)c, yellow arrow) and also showed lymphocytic infiltration (Figure 5(B)c&e, black arrows). The lung tissue in certain areas with undefined carcinogenic features was seen to have apoptotic regions and minimal lymphocytic infiltration (Figure 5(B)d, black arrows). Also, no systemic toxicity was observed upon therapy as seen in liver, kidney and spleen tissues (Figure 5(B) e–g). The tumor lesions identified grossly were confirmed to have tumorigenic origins by flow cytometric identification of EpCAM^+^ and CD45^+^ cell populations in the excised tumor lesions single cell suspensions. EpCAM is an epithelial cell marker essential to identify adenocarcinoma whereas CD45 is a lymphocytic marker and can indicate an inflammatory background induced due to urethane. The excised tumors displayed EpCAM^+^ cells population (29.6%) and CD45^+^ cells population (44.8%) (Figure 5(C)a,b). This demonstrated that the sample had lymphocytic infiltration as seen from the histological examinations. This screening helped in identifying that the gross tumor nodule on the lung tissue was of cancerous nature and therefore was used further for cytometric analysis.

### Effects on apoptosis, ROS generation and mitochondrial potential

**Figure 6.**
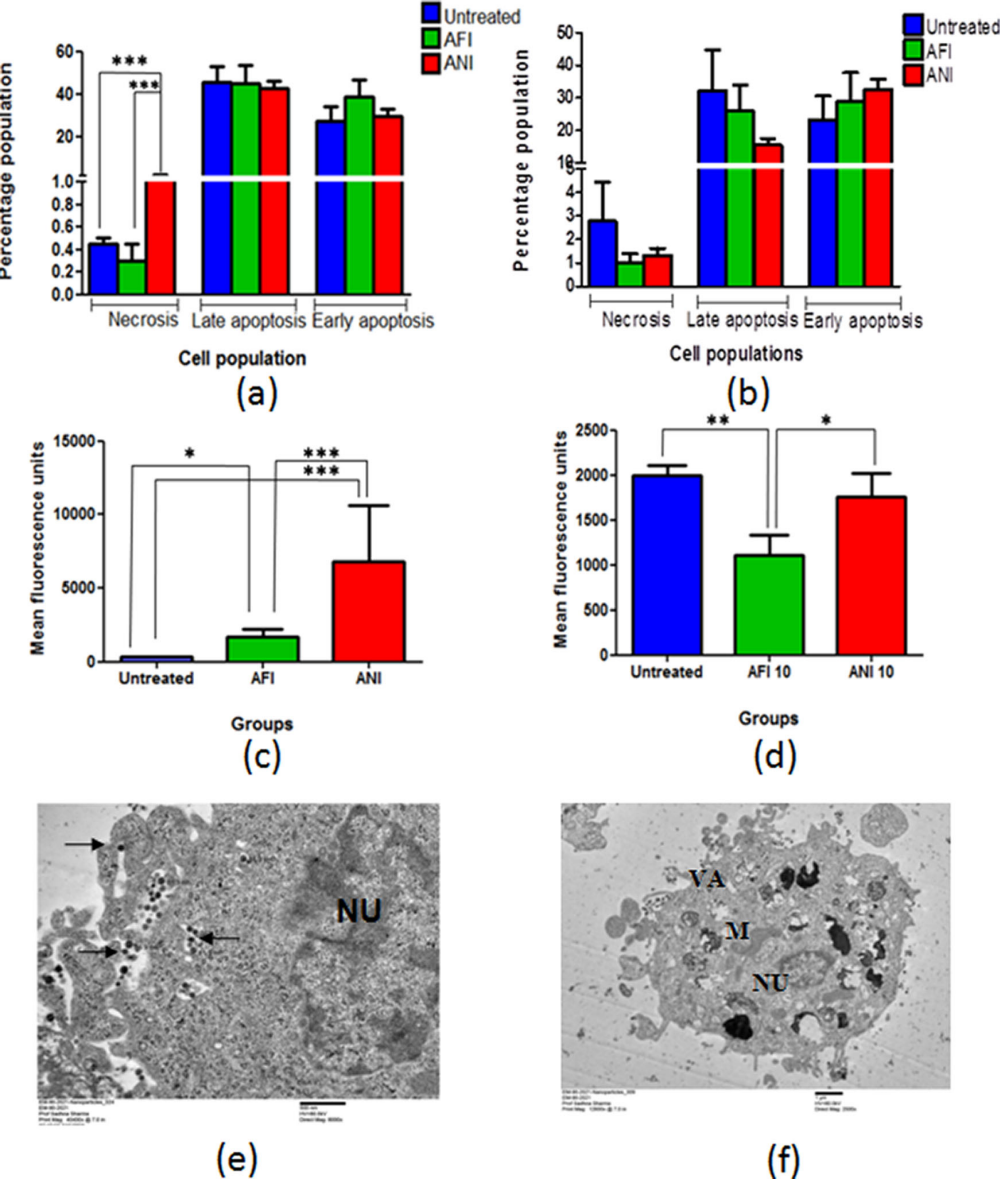
Flow cytometric analysis of lung adenocarcinoma lesions. Percentage of population representing necrosis and apoptosis in actinonin-treated animal group of (a) 6 months latency period and (b) 10 months latency period. The mean fluorescence units representing ROS levels in actinonin treatment group of (c) 6 months latency period and (d) 10 months latency period. (e) TEM image of NCI-A549 cellular uptake of actinonin nanoparticles. (f) TEM image of NCI-A549 cells exposed to actinonin nanoparticles for 48 h. Values are mean ± S.E. of 3–5 animals. UT – untreated, AFI – free actinonin i.p and ANI – nanoformulated actinonin i.p. ***p ≤ 0.01; **p ≤ 0.01; *p ≤ 0.05.

In the actinonin-treated 6 months latency period mice model, the necrotic cell population was high as compared to the untreated group, while no change was observed in the late apoptotic cell population of the residual tumor lesions (Figure 6a). The animals treated with free and nanoformulated actinonin had lower late apoptotic and necrotic cell populations in the remnant/residual tumor lesions as compared to untreated mice in 10 month latency period model (Figure 6b). The *ex vivo* TEM based experiments on NCI-A549 human lung adenocarcinoma cells demonstrated the actinonin nanoparticles treated cells with apoptotic features like cellular blebbing, loss of mitochondrial integrity, numerous vacuoles and shrunken nucleus (Figure 6e,f). The cancer cells have a tendency to scavenge the reactive oxygen species in order to cytoprotect and maintain their proliferative potential. The 6-month latency period model upon treatment with free and nanoformulated actinonin showed increased ROS levels in the remaining tumor lesions in contrast to 10 month latency model (Figure 6a,b). Therefore, actinonin-treated animals showed varied effects on ROS production and could be latency period dependent.

**Figure 7.**
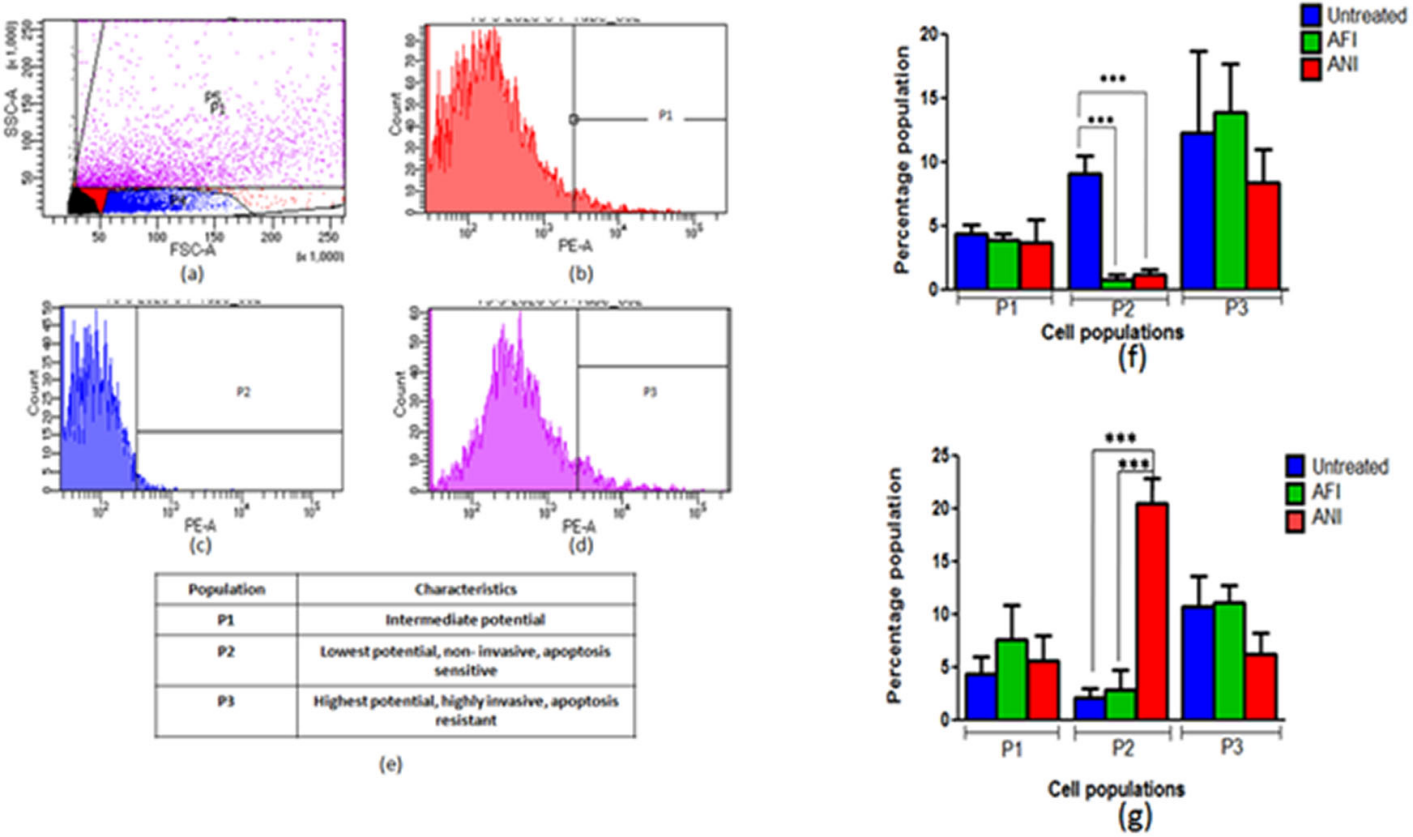
Selection of population based on mitochondrial membrane potential. (a) Scatter plot with all subpopulations, (b) cells with higher to intermediate fluorescence intensity (P1), (c) cells with lowest fluorescence intensity (P2), (d) cells with highest fluorescence intensity (P3), (e) tabulated subpopulations with mean fluorescence intensities and percentage in parent population. The percentage population representing TMRM positivity in (f) actinonin treatment animal group of 6 months latency period and (g) actinonin treatment animal group of 10 months latency period. UT – untreated, AFI – free actinonin i.p and ANI – nanoformulated actinonin i.p. Values are mean ± SE of 3–5 animals. ***p ≤ 0.01.

The cancerous cells demonstrate higher mitochondrial membrane potential than the normal cells. Therefore, to estimate the effect of treatment on the tumors, treatment group tumor cell suspensions were incubated with TMRM, a potentiometric dye and were quantified using flow cytometer (Figure 7).

The animals when treated at the 6 months latency period showed lower levels of P2 &P3 cell subpopulations in free and nanoformulated actinonin (Figure 7f). When the 10-month latency group was treated with actinonin nanoformulation, the subpopulation P3 showed lower levels unlike P2 cell populations (Figure 7g). Thus overall, the actinonin nanoformulation had comparable effects on the tumor lesions when compared with its free form. The therapeutic efficacy of the treatment was further evaluated at the genetic level to correlate with the therapeutic outcomes in terms of tumor burden and survival.

**Figure 8.**
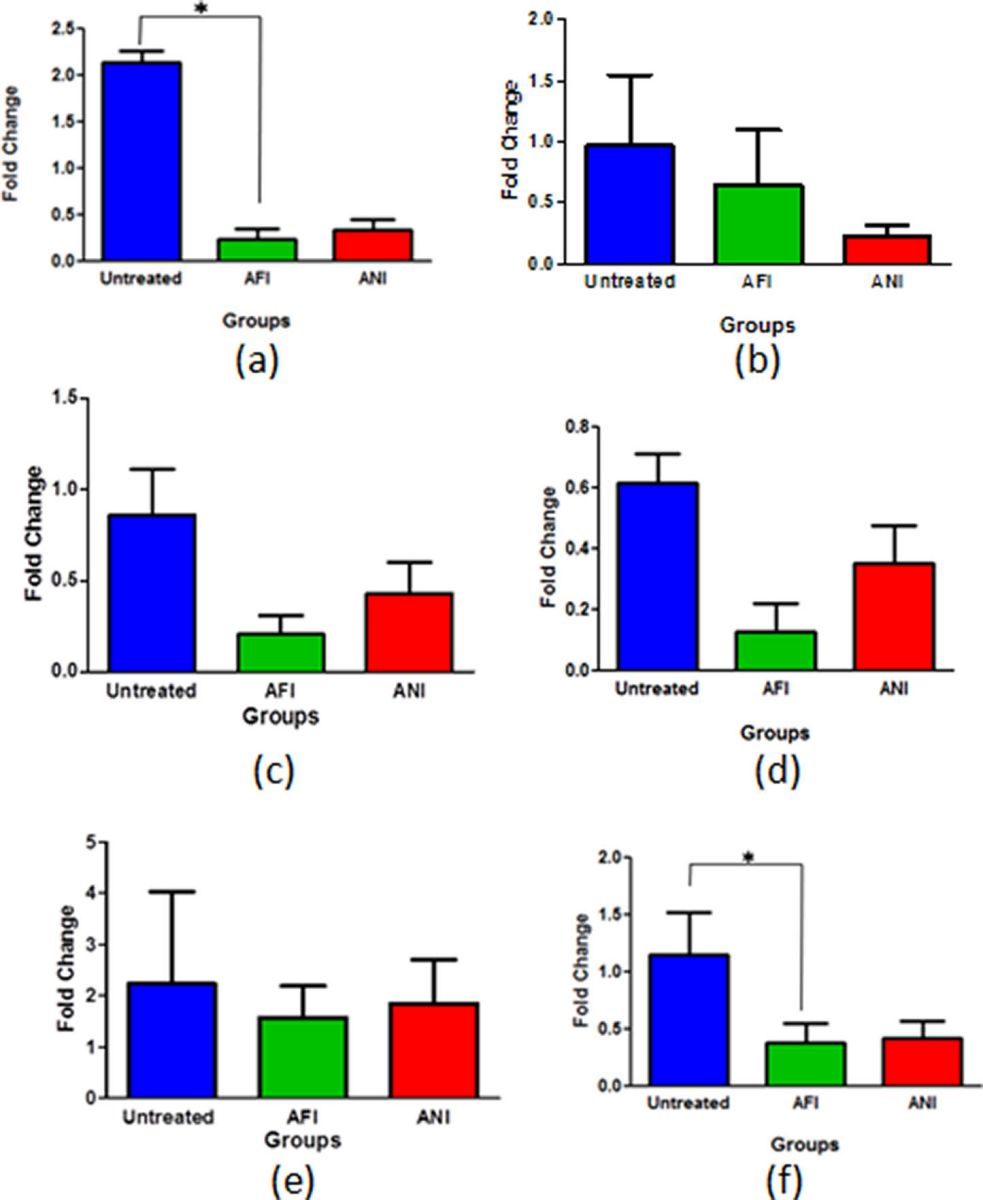
Gene expression analysis of lung adenocarcinoma lesions. The *egfr* mRNA levels at (a) 6 months latency period and (b) 10 months latency period. The *folR α* mRNA levels at (c) 6 months latency period, (d) 10 months latency period. The *pdf* mRNA levels at (e) 6 months latency period (f) 10 months latency period. UT – untreated, AFI – free actinonin i.p. and ANI – nanoformulated actinonin i.p. Values are mean ± SE of 3–5 animals *p ≤ 0.05.

Actinonin-treated groups in its free and nanoformulated forms showed considerable decrease in *egfr* expression in the 6 and 10 months latency period mice model (Figure 8a,b). The folate receptor α is highly expressed in lung cancers and as the formulation was targeted toward the folate receptors, it was evident to study its expression in various groups to estimate the effect on tumor cell burden. All the treatment groups had decreased expression of folate receptor α when compared with untreated groups in both 6 and 10 months latency period mice model (Figure 8c,d). Actinonin-treated groups both in its free and nanoformulated forms showed down-regulation in peptide deformylase expressions in both latency models (Figure 8e,f).

## Discussion

The present study evaluated an alternative strategy in order to improve the antiproliferative potential of actinonin. Folate as a targeting moiety and human serum albumin as a biocompatible nanocarrier were utilized for the delivery of the drug. The physical characterization of the nanoparticles was consistent with the attributes of a nanosuspension. The pharmacokinetic studies demonstrated sustained release profile of actinonin nanoformulation when administered intraperitoneally. Therefore, the pharmacokinetic evaluation of nanoformulation encouraged its comparison with its free drug form for its dose frequency as well as its therapeutic efficacy. As per expectations, the nanoformulated actinonin displayed comparable/improved therapeutic effects in terms of survivability, reduced tumor burden and downregulated tumor markers’ expression levels.

Considering the lack of sufficient literature on actinonin’s *in vivo* anti-cancerous effects, the present experimental work is of critical importance. In a solid tumor, heterogeneous cell populations can be differentiated upon mitochondrial membrane potential. Loss in P2 population clearly reflects positive effects of therapy which may be aided by ROS generation. At the 10 months latency period, induction of necrosis by the nano actinonin group was observed which has not been reported so far in the limited literature available, specifically on actinonin’s mechanism of action. As the current study is first of its kind to evaluate the apoptotic status of *in vivo* urethane-induced lung adenocarcinoma in treated mice, therefore effects of actinonin need further exploration with respect to *in vivo* models. Further, as per anticipation, actinonin in its free and nanoformulated form intraperitoneally showed downregulation in *egfr* mRNA levels. This can be contributed by meprin α which is inhibited by actinonin, which is shown to be involved in the chronic inflammation by activating IL-6 trans-signaling (Arnold et al., 2017; Wculek et al., 2020; ). IL-6 as a proinflammatory cytokine promotes inflammation thereby contributing in worse prognosis of NSCLC (Silva et al. 2017). Also, activated *EGFR* and ERK1/2 signaling via ligand shedding pathway may support tumor cell proliferation, migration as well as invasion (Minder et al., 2012). Overall experimental evidence indicate that actinonin may have a potential indirect inhibitory effect on *egfr* expression. Similarly, folate receptor α expression was considerably downregulated in groups treated with actinonin as free and nanoformulation for both latency groups. Also, folate receptor α has better discriminatory ability to distinguish between adenocarcinoma and squamous cell carcinoma (Nunez et al., 2012). Its downregulation indicates therapeutic benefits of treatment regimens of nanoformulated and free actinonin. Next, mitochondrial effector gene such as peptide deformylase was evaluated. In 10 months latency model, free and nano actinonin, however, showed more pronounced downregulation of *pdf* than the 6 months latency period model. These findings established the benefits of incorporation of folate conjugated actinonin nanoformulation as an improved and reliable chemotherapeutic option against lung adenocarcinoma as it demonstrated aforementioned anti-cancerous effects in low dose frequency as compared to actinonin in free form through intraperitoneal administration.
